# The Nature, Causes, and Clinical Impact of Errors in the Clinical Laboratory Testing Process Leading to Diagnostic Error: A Voluntary Incident Report Analysis

**DOI:** 10.1097/PTS.0000000000001166

**Published:** 2023-09-28

**Authors:** Christel van Moll, Toine Egberts, Cordula Wagner, Laura Zwaan, Maarten ten Berg

**Affiliations:** From the ∗ Department of Internal Medicine, University Medical Center Utrecht; † Utrecht Institute for Pharmaceutical Sciences and Division of Pharmacoepidemiology and Clinical Pharmacology, Faculty of Science, Utrecht University; ‡ Department of Clinical Pharmacy, University Medical Center Utrecht; § Netherlands Institute of Health Services Research (NIVEL), Utrecht; ∥ Amsterdam Public Health institute (APH), Amsterdam University Medical Center, Amsterdam, the Netherlands; ¶ Erasmus Medical Center, Institute of Medical Education Research Rotterdam, Rotterdam, the Netherlands; ∗∗ University Medical Center Utrecht, Central Diagnostic Laboratory, Utrecht, The Netherlands.

**Keywords:** diagnostic error, patient safety, incident reports, laboratory medicine

## Abstract

**Objectives:**

Diagnostic errors, that is, missed, delayed, or wrong diagnoses, are a common type of medical errors and preventable iatrogenic harm. Errors in the laboratory testing process can lead to diagnostic errors. This retrospective analysis of voluntary incident reports aimed to investigate the nature, causes, and clinical impact of errors, including diagnostic errors, in the clinical laboratory testing process.

**Methods:**

We used a sample of 600 voluntary incident reports concerning diagnostic testing selected from all incident reports filed at the University Medical Center Utrecht in 2017–2018. From these incident reports, we included all reports concerning the clinical laboratory testing process. For these incidents, we determined the following: nature: in which phase of the testing process the error occurred; cause: human, technical, organizational; and clinical impact: the type and severity of the harm to the patient, including diagnostic error.

**Results:**

Three hundred twenty-seven reports were included in the analysis. In 77.1%, the error occurred in the preanalytical phase, 13.5% in the analytical phase and 8.0% in the postanalytical phase (1.5% undetermined). Human factors were the most frequent cause (58.7%). Severe clinical impact occurred relatively more often in the analytical and postanalytical phase, 32% and 28%, respectively, compared with the preanalytical phase (40%). In 195 cases (60%), there was a potential diagnostic error as consequence, mainly a potential delay in the diagnostic process (50.5%).

**Conclusions:**

Errors in the laboratory testing process often lead to potential diagnostic errors. Although prone to incomplete information on causes and clinical impact, voluntary incident reports are a valuable source for research on diagnostic error related to errors in the clinical laboratory testing process.

Diagnostic errors are a common cause of iatrogenic harm to patients and are defined as a diagnosis that was wrong, delayed, or missed or was communicated with relevant delay to the patient.^1^ Recent data from the Netherlands showed that 11% of the adverse events that occurred in patients who died in the hospital were diagnostic errors and that 40% of severe incidents in hospitals that were reported to the Dutch Healthcare Inspectorate were related to errors in the diagnostic process.^2,3^ Diagnostic errors are also internationally considered to be a major patient safety problem. The National Academy of Medicine in the United States for example concluded in their influential report on improving diagnosis in health care that most people will experience a diagnostic error at least once in their lifetime.^4^

Clinical laboratory testing is a major component of the diagnostic process. Up to 80% of medical decisions are influenced by laboratory data.^5^ Errors in the laboratory testing process can lead to diagnostic errors. Gandhi et al^6^ found for example that in 37% of diagnostic errors in the ambulatory setting that led to malpractice claims, incorrect interpretation of the laboratory test result caused the error. Similarly Schiff et al^7^ found in a survey among physicians that errors occurred most frequently (44%) in the diagnostic testing phase (failure to order, report, and follow-up laboratory results).

Although the consequences for patients of errors in the laboratory testing process are well known from these studies, there are no studies in which diagnostic error as an outcome of an error in the laboratory testing process was investigated. Such knowledge could be useful in developing targeted risk management strategies to effectively improve the safety of diagnostic testing.

The process of clinical laboratory testing can be subdivided into the preanalytical, analytical and postanalytical phase. The preanalytical phase being all the steps occurring before the specimen is being analyzed, for example, ordering the test, collecting the specimen, and transport of the specimen. The analytical phase is the specific analysis of the specimen in the laboratory. The postanalytical phase covers the steps occurring after the specific specimen has been analyzed, that is, reporting the result to the clinician or patient and interpretation of the result by the clinician.^8^ Over time, there has been an interesting shift in which phase of the laboratory testing process most errors occurred. In the 1980s, most errors occurred in the analytical phase. This changed to the preanalytical phase 20 years later, when it was estimated in studies with only data on frequencies of type of laboratory errors that 60% of the errors occur in the preanalytical phase.^9–11^ Automation, improved laboratory technology, assay standardization, and better internal and external quality control majorly contributed to quality improvement and reduction of errors in the analytical phase.^11^

To prevent patients from harm through diagnostic errors, it is important to investigate whether there are strategies to help mitigate the risk for an error to occur. As Zwaan et al^12^ recently stated in their article on the current state of research on diagnostic errors, priority should be given to identification of the main failure points in the diagnostic process and in developing, implementing, and testing specific interventions. For this type of research, several types of data sources can be used, like claims data, obduction data reports on major incidents, and voluntary incident reports. This type of reports are an interesting source compared with the others because it provides an extensive view on the clinical process and an insight into the part of incidents that healthcare workers consider relevant and that reflect situations that have the potential to become major incidents.

Databases with voluntary incident reports have not been widely used yet for investigating errors in laboratory testing process. We are familiar with 3 large studies, performed in the United States and Canada.^13–15^ In the first study, an analysis of 37,532 laboratory event reports, it was found that most errors occurred in the preanalytical phase (specimen not labeled/mislabeled and improper collection) and that most errors did not cause harm.^13^ In the second study, an analysis of 12,278 laboratory related safety events reported to the British Columbia Patient Safety and Learning System, comparable results were found. Most incidents concerned the preanalytical phase (76%), and it was found that the majority of incidents (95.9%) resulted in little or no harm and 0.44% in severe harm.^14^ In that study, the association between the degree of harm and the phase of testing was investigated. It was found that postanalytical events contain the highest risk to result in severe harm.^14^ In those studies, it was not specifically investigated whether the incidents led to (potential) diagnostic errors. The third study from the same group with data from British Columbia Patient Safety and Learning System confirmed the findings from their first study.^15^ To gain more insight into diagnostic errors related to errors in the clinical laboratory testing process, we conducted a retrospective analysis of voluntary incident reports in our hospital, a large academic teaching hospital with high volume diagnostic testing (e.g., approximately 500,000 orders for clinical chemistry testing annually) in which we looked at the phases of the testing process, the type of errors, the causes and the clinical impact, including potential diagnostic errors, as well as the relation between these.

## METHODS

### Study Design

We conducted a retrospective study with reports concerning any type of incident concerning the clinical laboratory process of clinical genetics, medical microbiology, clinical chemistry, pathology, and metabolic diagnostics from the voluntary patient safety incident report system of the University Medical Center Utrecht (UMC Utrecht), a 1024-bed academic teaching hospital in the Netherlands.

### Voluntary Incident Report System

The voluntary patient safety incident report system that was used at the UMC Utrecht during the study period was the Incident Management System from the Patient Safety Company (Alkmaar, the Netherlands).

The system was online accessible through the internal Web site of the hospital. All personnel of UMC Utrecht can report any incidents related to patient care including hazards, near misses, and adverse events. Reports are filed using a standardized format for what happened where and when. The reporter has to describe the nature of the event and the consequences for the patient in free text.

The reporter must assign one of the following categories to the incident: medication, falls, behavior, information and communication technology, medical devices, the online patient portal, or other. Incidents in the category other are assigned labels by the central incident registration of the hospital based on the description of the incident by the reporter. One of the additional labels is diagnostics that is used for incidents related to any type of diagnostic testing.

Incidents will be underreported, as small incidents will happen and personnel will not be aware that this can be reported to improve the workflow. However, personnel working at the laboratory is being trained to report all incidents as part of daily work.

The incident reports are used for trend monitoring and improvement of workflow and the quality of patient care. Data are recorded and are reviewed weekly by independent health-care workers, to check if any high-impact incidents have happened that need additional actions. The system can be queried on characteristics to reports of interest for analysis.

### Data

For this study, we queried the system for all reports labeled as diagnostics (regardless of where the reported error occurred) and all reports from all other categories for which was reported that the error occurred at a clinical laboratory in 2017 and 2018. Of all these reports, for convenience purposes (limited time and the estimation we did not need all the reports for a representative analysis), the first 600 reports (in order of time of reporting) were included. From these we selected reports concerning the clinical laboratory process of clinical genetics, medical microbiology, clinical chemistry, pathology, and metabolic diagnostics for the analysis.

### Outcomes

Per report, we determined the nature, the cause, and the clinical impact of the error.

Before the study, the authors C.M., T.E., and M.B. established a 36-step classification scheme for the nature (type of error) of errors in the clinical laboratory testing process based on previously used classifications in order studies (Table 1).^10,16,17^ All 600 reports were read by 2 authors (C.M. and M.B.) and together they classified the nature of the incident into the 36 steps of laboratory process. For the classification of the causes, the Eindhoven classification model^18^ was used. For classification of the clinical impact, the classification previously used by Graber et al^19^ for diagnostic error was used. Two authors (C.M. and M.B.) classified the first 50 cases together, to determine how the different categories should be classified. Subsequently, C.M. classified the cases alone. In case of doubt, C.M. and M.B. discussed and decided together.

**TABLE 1:** Nature of Errors: 36 Steps of the Laboratory Testing Process

Main Phases of Laboratory Process	Subprocess	Type of Error in Subprocess	% of Events (n)
	Error in ordering test by clinician	Delayed order	0.6 (2)
		Wrong order	8.9 (29)
		No order	2.4 (8)
	Error in providing order to patient	Delay in providing the order	0.6 (2)
		Wrong patient or wrong form	0.3 (1)
		Not providing the order to the patient	0.6 (2)
	Error in processing the order	Delay in processing the order	0 (0)
		Wrongly processing the order	1.5 (5)
		Not processing the order	1.2 (4)
	Improper collection of specimen	Delay in collection	2.4 (8)
		Incorrect patient ID	1.5 (5)
Preanalysis		Improper specimen	0.6 (2)
		Improper container	7.3 (24)
		Improper collecting conditions	4.3 (14)
		Improper collection of specimen	0.9 (3)
		Problems in labeling the container	2.1 (7)
		Not collecting the specimen	6.1 (20)
	Error in transport of the specimen	Delay in transport	4.3 (14)
		Improper way of transport	1.2 (4)
		Not transporting the specimen	2.1 (7)
	Error in preanalytical steps in the laboratory	Delay in processing	6,4 (21)
		Swapping tests	1.5 (5)
		Incorrect patient ID	1.5 (5)
		Error in processing	3.1 (10)
		Specimen lost	8.0 (26)
	Unknown		6,1 (20)	
	Analytical error		8,0 (26)
Analysis	Error in analysis by laboratory specialist.		2,1 (7)
	Laboratory device unavailable		1.5 (5)
	Unknown		1.8 (6)
	Error in reporting result to clinician	Delay in reporting	1.2 (4)
		Incorrect results reported	3.1 (10)
		Not reporting results	0.9 (3)
	Not reading the result by clinician		0.6 (2)
	Misinterpretation of result by clinician		0.3 (1)
Postanalysis	Error in communication of result by clinician to patient	Delay in communication	0.3 (1)
		Incorrect result communicated	0.9 (3)
		Not communicating the result to the patient	0 (0)
	Unknown		0.6 (2)
Unclassifiable			1.5 (5)
Other			1.2 (4)

As incident reports describe the whole spectrum of incidents (near misses, minor errors with no consequences and diagnostic errors, etc.), we defined the outcomes as followed:

- The nature of the error: In which phase of the laboratory testing process the error occurred was analyzed by using both the known 3-phase model of the laboratory testing process (preanalytical, analytical, and postanalytical) and by the 36-step classification scheme (Table 1).
- The cause of the error: Full free-text descriptions of the incident reports were examined for causes based on the classification used in the periodical Dutch report on health-related harm.^2^ Causes were grouped into one of the following 3 main categories: human factors, technical factors, and organizational factors. Per report, we only scored the most prominent cause.
- The clinical impact of the error: This includes the severity (including the event resulted in a diagnostic error) and the consequences of the error. Severity was classified into the following categories: unknown, no harm, distress/anxiety, new sample taken, potential delay in diagnosis or treatment due to a late test result, a missed diagnosis, and a wrong diagnosis. The latter three were considered as a potential diagnostic error, as defined by Graber et al.^1^ The consequences of the error were classified into the following types: no harm (no negative consequences for the patient at all), minor harm (patient inconvenience), moderate harm (i.e., delay in surgery schedule, new sample taken), major harm (i.e., wrong treatment started based on wrong laboratory result), disastrous (i.e., death, permanent disability, or near life-threatening event), and unknown.

## RESULTS

At the UMC Utrecht, a total of 11,827 patient safety incidents were reported in 2017 and 2018.

Of these, 949 (8%) were labeled as related to diagnostic testing, including laboratory diagnostics, radiology and function testing like electrocardiogram and lung function testing.

**FIGURE 1:**
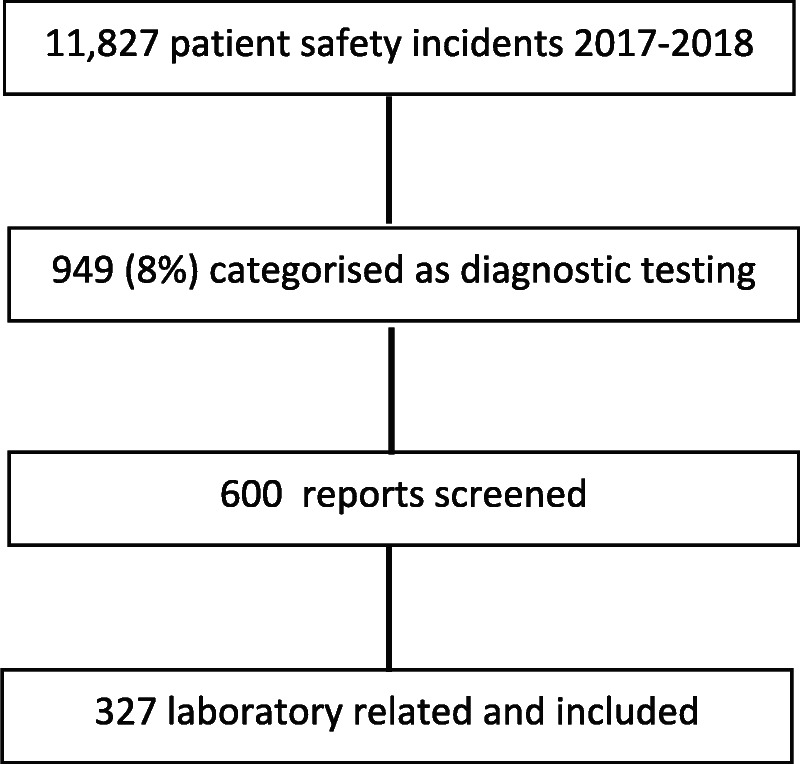
Flow chart selection of incident reports.

The first 600 reports in time (of the 949) were screened for being related to the laboratory testing process, which resulted in 327 reports, which were included in the analysis (Fig. 1). A total of 34.5% of these reports (n = 113) were reported by laboratory workers. Most errors (45.3%, n = 148) occurred in the clinical ward, compared with occurrence in the laboratory setting.

For illustration purposes, we provide 3 examples of voluntary reports with the data we extracted in Table 2.

**TABLE 2:** Examples of Incident Reports on Errors in the Laboratory Testing Process (for Illustrative Purposes)

Incident Description	Nature (Phase of the Testing Process)	Cause	Clinical Impact	Diagnostic Error
The laboratory results of this baby of 04-12-2017 showed a lipase of 858 mmol/L. The day before the result was 51 mmol/L. Because of a suspected pancreatitis, hyperhydration was started. On 05-12-2017, an abdominal ultrasound was performed, which showed no signs of pancreatitis, the laboratory results showed a lipase of 41 mmol/L. The results of 04-12-2017 were reanalyzed and showed a lipase of 28 mmol/L.	Analysis—analytical error	Unidentifiable	Wrong diagnosis, major harm (extra diagnostic test, wrong therapy started).	No
Tube for determining the blood type was lost, after arriving at the laboratory. We searched the laboratory and called the ward, but the tube could not be found. The blood type could not be determined. The blood must be drawn again.	Preanalytical phase—error in preanalytical steps in the laboratory—specimen lost.	Human	Wrong diagnosis, unidentifiable	Yes
Wrong leucocyte differentiation results were reported in this patient. Laboratory worker 2 checked the results of her colleague, laboratory worker 1, and noticed that the wrong results were reported in the system.	Postanalysis—error in reporting result to clinician—wrong results reported	Human	Wrong diagnosis, unidentifiable	Yes

### Nature of the Error

In Table 1, the distribution of the errors over the phases of the laboratory testing process is presented. Most errors occurred in the preanalytical phase, 77.1% (n = 252). Analytical and postanalytical errors accounted for 13.5% (n = 44) and 8.0% (n = 26), respectively. A total of 1.5% of laboratory errors (n = 5) could not be classified. The top 3 category of error types were improper collection of the specimen (n = 83, 25.4%), errors in preanalytical steps in the laboratory (n = 67, 20.5%), and errors in ordering the test by the clinician (n = 39, 11.9%).

### Cause of the Errors

In Table 3, the distribution of the errors by cause category is presented. Human factors were the most frequent causes (58.7%, n = 192). Technical causes accounted for 12.5% (n = 41) and organizational factors accounted for 14.4% (n = 47). In 14.4% (n = 47), a cause could not be determined. Human and organizational errors were most prevalent in the preanalytical phase. Errors in the analytical phase were mostly due to technical issues.

**TABLE 3:** Causes of Errors in the Laboratory Testing Process

		Causes			
		Human, % (n)	Organizational, % (n)	Technical, % (n)	Unidentifiable, % (n)
Nature of the errors: main phases of the laboratory testing process				
Preanalysis		85.4 (164)	85.0 (40)	29.2 (12)	76.6 (36)
Analysis		5.2 (10)	8.5(4)	65.9 (27)	6.4 (3)
Postanalysis		8.8 (17)	6.4(3)	4.9 (2)	8.5 (4)
Unknown		0.5 (1)	0.0 (0)	0.0 (0)	8.5 (4)
Total % (n)		58.7 (192)	14.4 (47)	12.5 (41)	14.4 (47)

### Clinical Impact of the Errors

Table 4 presents the distribution of the errors by severity and the consequences of clinical impact, including diagnostic errors. In total 195 cases (59.6%), the error in the laboratory testing process resulted in diagnostic error. Of these, diagnostic errors with a highly negative consequence on the patient outcome ([4] major impact and [5] disastrous impact) accounted for 7.6% (n = 25) of all laboratory testing error reports. Of the 195 potential diagnostic errors, 32.1% concerned misdiagnosis, 17.3% missed diagnosis, and 50.5% delay diagnostic process.

**TABLE 4:** Clinical Impact of Patient Outcome: Number of Errors in the Laboratory Testing Process, Severity Versus Consequences, Including Diagnostic Errors

		Severity of Patient Outcome						
		No harm (%)	Distress (%)	New Sample (%)	Delay in diagnostic process (%)	Missed Diagnosis (%)	Misdiagnosis (%)	Unknown (%)
Consequences					
No		28 (8.6)	0 (0.0)	0 (0.0)	4 (1.2)	5 (1.5)	20 (6.1)	0 (0.0)
Minor		0 (0.0)	6 (1.8)	56 (17.1)	26 (8.0)	12 (3.7)	10 (3.1)	1 (0.3)
Moderate		0 (0.0)	6 (1.8)	22(6.7)	50 (15.3)	9 (2.8)	17 (5.2)	0 (0.0)
Major		0 (0.0)	0 (0.0)	0 (0.0)	6 (1.8)	4 (1.2)	11 (3.4)	0 (0.0)
Disastrous		0 (0.0)	0 (0.0)	0 (0.0)	3 (0.9)	0 (0.0)	1 (0.3)	0 (0.0)
Unidentifiable		0 (0.0)	1 (0.3)	1 (0.3)	9 (2.8)	4 (1.2)	4 (1.2)	11 (3.4)

Table 5 presents the distribution the different phases of the laboratory testing process and the consequences of the errors. Most errors occurred in the preanalytical phase (77.1%). Most errors occurring in the preanalytical phase had no to moderate consequences.

**TABLE 5:** Nature; Main Phases of the Laboratory Testing Process Versus Severity of Patient Outcome

		Consequences of Patient Outcome						
		No, % (n)	Minor, % (n)	Moderate, % (n)	Major, % (n)	Disastrous, % (n)	Unidentifiable, % (n)	Total
Nature: main phases of the laboratory testing process			
Preanalysis		17.5 (44)	38.1 (96)	32.5 (82)	3.9 (10)	0.0 (0)	7.9 (20)	77.1 (252)
Analysis		20.5 (9)	18.2 (8)	31.8 (14)	11.4 (5)	6.8 (3)	11.4 (5)	13.5 (44)
Postanalysis		15.4 (4)	19.2 (5)	19.2 (5)	23.1 (6)	3.8 (1)	19.2 (5)	8.0 (26)
Unidentifiable		0 (0)	40.0 (2)	60 (3)	0 (0)	0 (0)	0 (0)	1.5 (5)

Most errors with severe clinical impact occurred in the analytical and postanalytical phase, 32% and 28%, respectively. Errors occurring in the postanalytical phases had major to disastrous consequences in 26.9% of cases.

## DISCUSSION

This study provides insights into the nature, causes, and clinical impact of errors in clinical laboratory testing. To our knowledge, this is the first study with voluntary incident reports concerning errors in laboratory testing in which the clinical consequences of the error and its association with the type of error were investigated. We found that preanalytical errors, which are most frequent, have relatively mild consequences for the patients and analytical and postanalytical errors are less frequent but result in more harm to patients.

This finding is not unexpected. Preanalytical errors often result in a stop or a delay in the test process. Analytical and postanalytical errors take longer to be detected and result more often in a wrong diagnostic follow-up and subsequent treatment, potentially causing significant harm to the patient.

Our finding that most errors occur in the preanalytical testing phase (77.1%) is consistent with the results from the study by Snydman et al^13^ (81.1%) and the studies of group of Noble (69.1%).^14,15^ Preanalytical events range from 62% to 88% in other studies in which data from other sources than incident reports were used.^6,10,20^

In our study, 13.5% of the events are related to the analytical phase, and this is consistent with literature in which 11% to 18% of the events could be related to the analytical phase.^20,21^ Our number of laboratory errors in the postanalytical phase (8.0%) is comparable with the study by Snydman et al,^14^ in which 5.2% of all errors were postanalytical.

The analysis of the impact of the error on the patient consisted of different measures, the severity, and the consequences. A total of 59.6% of the investigated reports concerned a potential diagnostic error. A total of 77.1% of the potential diagnostic errors occurred in the preanalytical phase, but most errors in this phase had no to moderate consequences (88.1%). The rate of potential diagnostic errors with a high level of patient impact was low, 7.6% of all laboratory event reports. This rate is in line with literature, in which the risk of adverse events of inappropriate care due to laboratory errors ranges from 1.7% to 12%.^10^ Potential diagnostic errors with a high level of patient impact could mostly be related to the analytical and postanalytical phase. These data demonstrate that the analytical and postanalytical phases, in particular the reaction to aberrant laboratory results, is more important causes of potential adverse outcomes for patients, compared with errors in the preanalytical phase.^21,22^

The greater aim of our research is prevention of harm to patients due to diagnostic error. The results of the current study can assist in identifying were in the diagnostic process we should focus to gain the most impact. Errors in the laboratory testing process and their underlying causes are diverse, as are their consequences. In preventing these types of errors with a systemic approach different strategies can be chosen, one is to focus on the errors that have biggest clinical impact, that is, analytical and postanalytical errors. In our opinion, analytical errors, mainly caused by technical errors, are difficult to prevent, because laboratory tests are highly developed, controlled, and automated. Analytical errors are mainly causes by technical errors.

Although the number of postanalytical errors is relatively low their impact is high compared with preanalytical and analytical errors. Postanalytical processes, especially follow-up of test result, depend strongly on human actions in critical steps. In the past decades, the risk of lack of follow-up of test results has been identified as a major issue in patient safety. A closed-loop system in which test results are reported to the ordering physician in the electronic health record is a minimal requirement. However, these systems have to support the physician in such a way there is a minimal change to miss a test result of to forget to follow up the result. Recent literature shows that follow-up of test results is still a major health threat to patients. Lack of proper implementation of solutions and policies is identified as a cause.^23^

For investigating the nature, cause and impact of errors in the laboratory testing process different types of data sources can be used. In addition to voluntary incident reports, as we did, one could use databases with medical claims or major safety incidents, or medical chart review. These data sources will result in different findings concerning the severity of the impact of the incident. Incidents reported as voluntary incident report are less severe and will often concern near misses.^24^ The value of voluntary incident reports compared with data on major incidents and claims is that these reports provided a wider perspective of potential risk in the laboratory testing process where the other two are likely to represent the tip of the iceberg of incidents.

Incident report systems are an important tool to detect patient harm and failures in quality of clinical care. Since the release of the report “*To Err is Human*,” voluntary reporting systems have proliferated and are mandatory by, among others, the Joint Commission.^25,26^ However, it is still challenging to optimize these systems to collect appropriate data and to effectively improve patient care.^25^ A successful patient safety learning system must result in increased awareness of the risk in the work and in improved risk management. Important aspects of a successful system are systematic data analysis and feedback.^26^ To maximize the chance to generate meaningful learning outcomes, it is important to standardize and optimize (e.g., sufficient detail) the classifications used for type of error, the underlying causes, and clinical impact. With regard to the type of error in the laboratory testing process, our study provide a detailed classification, which recently has been implemented in the voluntary incident reporting system of our hospital.

A limitation of our study is lack of detailed data on causes (in 14.4% undetermined), patient outcomes (9.1% type of harm undetermined), and information on the patient care setting in incident reports.

This limited the classification of some of the reports, for example, the report generally focused on the main cause, and therefore it was not possible to analyze multiple causes per incident. Furthermore, because the incident reports are voluntary, it is likely that not all laboratory incidents were reported. However, there is no reason to believe that diagnostic errors are substantially more or less underreported than other type of errors. Nevertheless, our data do not allow to give an estimate of the incidence of laboratory errors.

## CONCLUSIONS

This study gained insight into the nature, outcomes, and clinical impact of errors in the laboratory testing process. Most errors occur in the preanalytical steps; however, errors in the analytical and postanalytical phases have more clinical impact. Errors in laboratory testing often lead to potential diagnostic errors, mostly a delay in the diagnostic process.

To be even a more useful source for investing safety of diagnostic testing, voluntary incident reports need to contain more detailed and standardized information, especially on cause and clinical impact.

Compared with previous estimates most errors still occur in the preanalytical phase, but we found these errors to have relative low impact on the patient outcome. The most harmful events were caused by human factors and occurred in the analytical and postanalytical phases. We think that these aspects of clinical laboratory diagnostics currently need the most attention in improving patient safety. Together with the new classification of the type of error in the laboratory testing process presented in this study, classification for cause and clinical impact can be implemented in voluntary incident report systems in hospitals. Wide adoption would provide valuable data in guiding efforts in further improving quality of care.
